# Inhibition of Protein Phosphatase 1 Stimulates Noncanonical ER Stress eIF2α Activation to Enhance Fisetin-induced Chemosensitivity in HDAC Inhibitor-resistant Hepatocellular Carcinoma Cells

**DOI:** 10.3390/cancers11070918

**Published:** 2019-06-29

**Authors:** Yi-Sheng Liu, Yu-Chun Chang, Wei-Wen Kuo, Ming-Cheng Chen, Hsi-Hsien Hsu, Chuan-Chou Tu, Yu-Lan Yeh, Vijaya Padma Viswanadha, Po-Hsiang Liao, Chih-Yang Huang

**Affiliations:** 1 Program for Aging, China Medical University, Taichung 404, Taiwan; 2 Division of Hematology and Oncology, Department of Medicine, Kaohsiung Armed Forces General Hospital, Kaohsiung 802, Taiwan; 3 Graduate Department of Biological Science and Technology, National Pingtung University of Science and Technology, Pingtung 912, Taiwan; 4 School of Chinese Medicine, China Medical University, Taichung 404, Taiwan; 5 Department of Biological Science and Technology, China Medical University, Taichung 404, Taiwan; 6 Department of Surgery, Division of Colorectal Surgery, Taichung Veterans General Hospital, Taichung 407, Taiwan; 7 Faculty of Medicine, National Yang-Ming University, Taipei 112, Taiwan; 8 Division of Colorectal Surgery, MacKay Memorial Hospital, Taipei 104, Taiwan; 9 MacKay Medicine, Nursing and Management College, Taipei 112, Taiwan; 10 Division of Chest Medicine, Department of Internal Medicine, Armed Force Taichung General Hospital, Taichung 411, Taiwan; 11 Department of Pathology, Changhua Christian Hospital, Changhua 500, Taiwan; 12 Department of Medical Technology, Jen-Teh Junior College of Medicine, Nursing and Management, Miaoli 356, Taiwan; 13 Department of Biotechnology, Bharathiar University, Coimbatore 641046, India; 14 Graduate Institute of Basic Medical Science, China Medical University, Taichung 404, Taiwan; 15 Cardiovascular research center, Hualien Tzu Chi Hospital, Hualien 970, Taiwan; 16 Center of General Education, Buddhist Tzu Chi Medical Foundation, Tzu Chi University of Science and Technology, Hualien 970, Taiwan; 17 Department of Medical Research, China Medical University Hospital, China Medical University, Taichung 404, Taiwan; 18 Department of Biotechnology, Asia University, Taichung 413, Taiwan

**Keywords:** hepatocellular carcinoma, fisetin, protein phosphatase 1, eIF2α, chemosensitivity

## Abstract

Hepatocellular carcinoma (HCC) is a common fatal type of malignant tumor that has highly metastatic and recurrent properties. Fisetin is a natural flavonoid found in various vegetables and fruits which exhibits anti-cancer and anti-inflammatory properties, as well as other effects. Thus, we hypothesized that fisetin can act as an adjuvant therapy in cancer or drug-resistant cancer cells, and further investigated the molecular mechanisms underlying the development of drug-resistance in HCC cells. We found that fisetin effectively inhibited the cell viability of not only parental cells but also histone deacetylase inhibitors-resistant (HDACis-R) cells and enhanced the chemosensitivity of HCC cells. Interestingly, fisetin did not induce cell apoptosis through the activation of the endoplasmic reticulum (ER) stress sensor of protein kinase R (PKR)-like endoplasmic reticulum kinase, but rather through the non-canonical pathway of the protein phosphatase 1 (PP1)-mediated suppression of eIF2α phosphorylation. Moreover, fisetin-induced cell apoptosis was reversed by treatment with PP1 activator or eIF2α siRNA in HCC cells. Based on these observations, we suggest that PP1-eIF2α pathways are significantly involved in the effect of fisetin on HCC apoptosis. Thus, fisetin may act as a novel anticancer drug and new chemotherapy adjuvant which can improve the efficacy of chemotherapeutic agents and diminish their side-effects.

## 1. Introduction

In recent years, hepatocellular carcinoma (HCC) has become the second-leading cause of cancer deaths in Taiwan [1,2] and the fourth-leading cause of cancer mortality worldwide [3]. The hepatitis B and C viruses are the most common risk factors leading to chronic liver inflammation and cancer, obesity, or diabetes-induced fatty liver disease, while other risks include exposure to vinyl chloride (used in making plastics) or anabolic steroids, as well as inherited diseases and alcohol abuse [4]. Recent studies have indicated that numerous types of cancer therapy, such as surgery, radiation therapy, chemotherapy, immunotherapy, hormone therapy, and stem cell transplant, are able to treat HCC. A single treatment is employed in some patients, however most individuals undergo a combination of treatments, such as surgery with chemotherapy or radiation therapy [5]. However, drug resistance usually occurs in chemotherapeutic agents in liver cancer cells, and is the major cause of chemotherapy failure in patients [6]. 

It is known that anticancer drugs lead to cancer cell apoptosis by activating pro-apoptotic regulators or suppressing survival factors [7]. However, molecular signaling pathways may influence the efficacy of chemotherapeutic drugs, often involving the modification of the activity of pro- and anti-apoptotic factors [8]. Moreover, the regulatory functions of protein kinases and phosphatases may also influence the efficacy of chemotherapeutic drugs, given that their activity ratio determines the phosphorylation state of key cell survival proteins [9,10]. According to these studies, protein kinases are important determinants of malignant cell apoptosis. Protein phosphatase-1 (PP1) is a protein phosphatase which plays essential roles in the regulation of cell physiology [11]. PP1 was recently found to dephosphorylate Protein kinase B (Akt) at T405 [12], and is also known to promote cancer cell survival by the regulation of p53 [13]. Additionally, a recent study indicated that the induction of cell death by chemotherapeutic agents is often correlated with phosphatase activation [14]. However, the anti-apoptosis and anti-tumor mechanisms of PP1 in human HCC are not clearly understood.

The modification of histone proteins on N-terminal tails, such as acetylation, is a post-translational modification. Post-translational modifications are important to control for chromatin structure and function, and highly affect DNA-related processes and gene expression [15]. Following these studies, cancer epigenetics such as histone acetylation (HAC) and histone deacetylation (HDAC) have been recognized and used to treat HCC [16]. HDAC inhibitors have been researched in relation to many cancers, such as prostate cancer, pancreatic cancer, small-cell lung cancer, and liver cancer [17]. Some studies have shown that HDAC inhibitors induced cell apoptosis and decreased cell viability in liver cancer [18], however more evidence is required to elucidate the molecular mechanism behind this effect and cancer cell resistance to HDAC inhibitors. 

Our previous studies used apicidin which is a novel histone deacetylase inhibitor (HDACi) that is derived from a fungal metabolite [19] to treat a liver cancer cell line, HA22T to create an apicidin-resistant HA22T (apicidin-R) cell line [10,20,21]. Moreover, we also obtained the apicidin-R cells also resistance to other HDAC inhibitor-soruberoylanilide hydroxamic acid (SAHA) and revealed the drug-resistance via regulated of cofilin-1 phosphorylation and translocation in liver cancer [22]. Additionally, other studies have demonstrated that complementary therapies can be used in addition to conventional treatment to reinforce the immune system, relieve symptoms of liver cancer, and enhance the effectiveness of conventional therapies [23]. Our previous studies showed that natural compounds can inhibit cancer cell proliferation and migration [24]; for example, thymoquinone was shown to inhibit the migration of colon cancer by suppressing prostaglandin E2-induced COX-2 activation [25], taiwanin E was found to suppress colon cancer cell migration via the p38 Mitogen-activated protein kinase (MAPK) pathway to reduce MMP-2/9 expression [26], and taiwanin C was found to inhibit oral cancer cell migration via GSK-3β activation to reduce β-catenin expression [27]. These studies indicate that natural compounds may be able to play an important role cancer treatment in combination with chemotherapy in HCC [28,29].

Flavonoids are a broadly distributed class of plant pigments that are regularly consumed in the human diet given their abundance [30]. Fisetin (3,3’,4’,7 tetrahydroxy flavone) is a bioactive flavonol molecule found in fruits and vegetables such as strawberry, apple, persimmon, grape, onion, and cucumber, and has multiple properties, such as antioxidant and anti-inflammatory activity [31]. Several studies have demonstrated the effects of fisetin against numerous diseases. Fisetin exhibits neurotropic, anticarcinogenic, and anti-inflammatory effects, as well as other effects beneficial to health. Recent studies have demonstrated that fisetin induces cell growth inhibition and apoptosis in human non-small cell lung cancer by increasing the levels of mitochondrial reactive oxygen species (ROS) and activating the ER stress pathway by MAPK activation [32]. 

Moreover, recent studies have suggested that the Ser/Thr protein phosphatase PP1 and its nonenzymatic co-factor growth arrest and DNA damage-inducible protein 34 (GADD34) can reverse the phosphorylation of phosphorylated eukaryotic translation initiation factor 2 subunit α (p-eIF2α), which is a downstream product of ER-associated kinase [33], and play a key role in the regulation of protein synthesis [34] and cell survival [35].

In this study, we hypothesized that fisetin may provide chemotherapeutic effects against liver cancer via the regulation of the ER stress pathway or the activation of protein phosphatases in vivo and in vitro. Our data show that fisetin enhanced chemosensitivity via the regulation of eIF2α phosphorylation by inhibiting PP1 expression without affecting the ER stress pathway in HCC resistance cells. These results indicate that fisetin could be a supplemental therapy for liver cancer patients with chemodrug resistance.

## 2. Results

### 2.1. Influence of Fisetin on Normal and Liver-Cancer Cells

**Figure 1:**
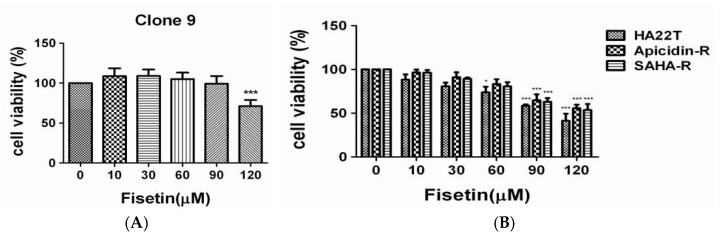
The effect of fisetin on normal cell and hepatocellular carcinoma cell viability. To explore the effect of fisetin on human liver cancer cell viability, we first treated normal cells (Clone 9 cells) and liver cancer cells (HA22T, AR, SR cells) with various concentrations of fisetin (10, 30, 60, 90, and 120 μM) for 24 h, and subsequently measured cell viability by 3-(4,5-dimethylthiazol-2-yl)-2, 5-diphenyltetrazolium-bromide (MTT) assay. (**A**) The results revealed a significant reduction of Clone 9 cell viability of approximately 70% following treatment with 120 μM fisetin for 24 h. (**B**) HA22T cell viability decreased in a dose-dependent manner after treatment with fisetin, especially for apicidin-R and soruberoylanilide hydroxamic acid resistant (SAHA-R) cells. The mean values were significantly different compared with the control group. ** *p* < 0.01; *** *p* < 0.001.

First, we assessed the effect of fisetin on the viability of normal and liver-cancer cells using the 3-(4, 5-dimethylthiazol-2-yl)-2, 5-diphenyltetrazolium-bromide (MTT) assay. Several concentrations of fisetin were used to evaluate cell viability after coculturing for 24 h. The results indicate that 10–90 μM fisetin did not reduce the cell proliferation of Clone 9 (Figure 1A), however it significantly attenuated the proliferation of HA22T parental cells, and especially AR and SR liver cancer cells in a dose-dependent manner (Figure 1B). Thus, fisetin could be used as a complementary therapy to enhance the effectiveness of chemotherapy.

### 2.2. Assessment of Fisetin as a Complementary Therapy in Hepatocellular Carcinoma Chemotherapy

**Figure 2:**
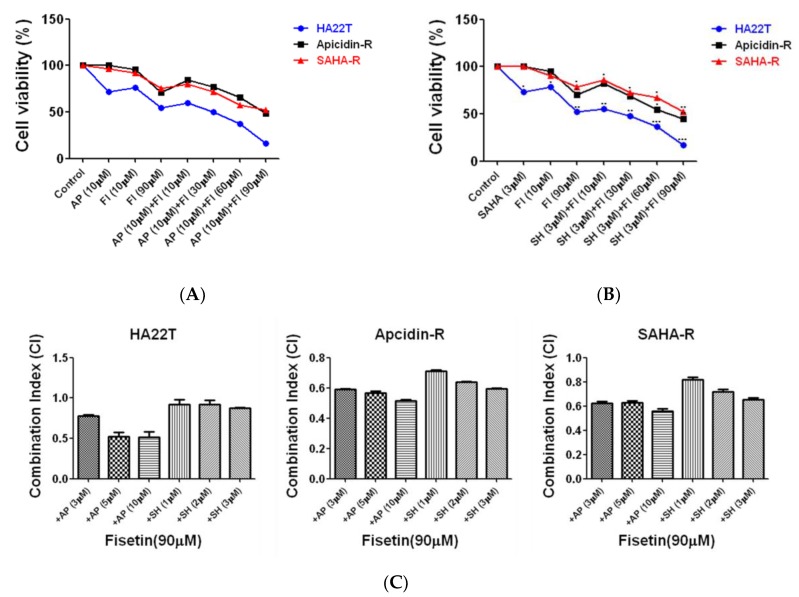
Fisetin enhances chemosensitivity in hepatocellular carcinoma (HCC) cells. Cell viability of HA22T, apicidin-R, and SAHA-R cells which were exposed to histone deacetylation (HDAC) inhibitors for 48 h and treated with fisetin at the 24th hour, as determined by MTT assay. (**A**,**B**) Liver cancer cell viability decreased in a dose-dependent manner after treatment with 10–90 μM fisetin together with a high dose of HDAC inhibitors. The data are expressed as a percentage of the control and are presented as the mean ± S.D. (* *p* < 0.05, ** *p* <0.01 and *** *p* < 0.001) difference between fisetin and the control group; mean ± S.D. *p* < 0.05, represents a significant difference between the fisetin-only treatment and the combination treatment group. (**C**) Combination index values for HDAC inhibitors and fisetin combinations for each liver cell line. Combination index values that are statistically significantly less than 1 indicate synergistic interactions, those that are statistically significantly more than 1 indicate antagonistic interactions, and those that are equal to 1 indicate additive interactions.

Combination chemotherapy has been shown to reduce the development of resistant cancer cells via different mechanisms [36]. In this study, we investigated the effect of fisetin in enhancing the effect of chemotherapy on HCC cells. HA22T, apicidin-R, and soruberoylanilide hydroxamic acid resistant (SAHA-R) cells were treated with HDACis (SAHA or apicidin) for 48 h, with fisetin being added at the 24th hour, and cell viability was subsequently measured by MTT assay. The results show that, in HCC cell lines, co-treatment with HDACi (apicidin 10 μM and SAHA 3 μM) and fisetin (10–90 µM) significantly reduced cell viability in a dose-dependent manner compared with the HDAC inhibitors alone (Figure 2A,B). Importantly, the obtained combination index (CI) [37] values (CI < 1) show that fisetin synergistically interacted with HDACi (Figure 2C), not only for parental cells but also resistance cell lines. These results show that fisetin can be used as a complementary therapy in cases of HDACi resistance by enhancing the chemosensitivity of HCC cells.

### 2.3. The ER Stress-Dependent Pathway Was not Significantly Involved in the Effect Of Fisetin on Liver Cancer Cells 

**Figure 3:**
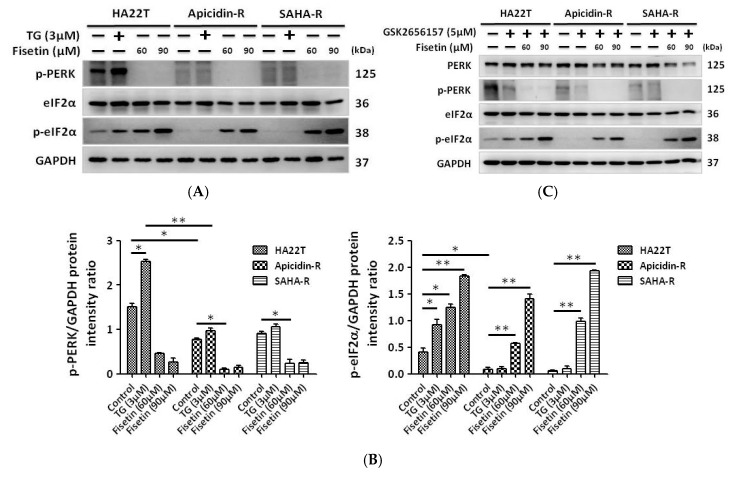

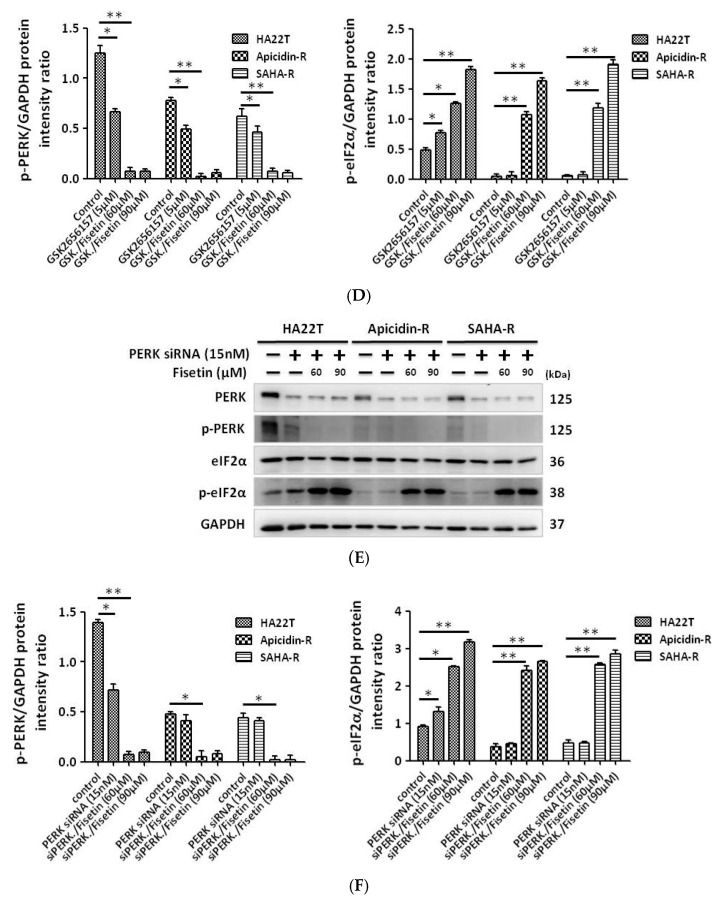
The ER stress molecular pathways cannot regulate eIF2α activation in HCC cells after treatment with fisetin. Cells were treated with thapsigargin (TG) or fisetin to determine whether the activation of phosphorylated eukaryotic translation initiation factor 2 subunit α (p-eIF2α) was regulated by PERK in HCC cells, and this activation was confirmed by GSK2656157 or small interfering RNA (siRNA). (**A**,**B**) The level of p-eIF2α in liver cancer cells was increased in a dose-dependent manner after treatment with fisetin without PERK activation. Western blotting data were quantified by densitometry and the ImageJ software, and normalized to glyceraldehyde 3-phosphate dehydrogenase (GAPDH). * *p* < 0.05, ** *p* < 0.01, *** *p* < 0.001 compared with the control group. (**C**–**F**) p-PERK knockdown by GSK2656157 or siRNA cannot reverse the effects of dephosphorylation on eIF2α. All protein samples were analyzed by western blotting. The protein expression was normalized to GAPDH. * *p* < 0.05, ** *p* < 0.01, *** *p* < 0.001 compared with the control group.

Several studies have demonstrated that fisetin induced cancer cell apoptosis through the production of reactive oxygen species and the activation of endoplasmic reticulum stress-dependent signaling pathways [38]. Therefore, we investigated the effects of fisetin on the activity of the ER stress pathway in HCC cells. The results showed that the expression of PERK, which is an up-stream gene related to ER stress, decreased in HDACis-R cells, and treatment with ER stress inducer thapsigargin (TG) [39] only activated ER stress proteins such as PERK and p-eIF2α in parental cells (Figure 3A). Interestingly, fisetin induced the production of eIF2α which is ER stress down-stream protein phosphorylation without PERK expression in HCC cells (Figure 3A,B). Based on these data, we attempted to confirm whether fisetin induced the activation of eIF2α not through PERK but rather by treatment with small interfering RNA (siRNA) or an inhibitor (GSK2656157). The results showed that levels of p-PERK (phosphorylated PERK) were reduced by treatment with siRNA (15 nM) or GSK2656157 (5 μM), however eIF2α was still activated by fisetin in hepatocellular carcinoma cells (Figure 3C–F). Our results indicate that fisetin did not activate eIF2α through PERK in HCC cells.

### 2.4. Fisetin Induced the Phosphorylation of eIF-2α via the Inhibition of PP1 Expression in HCC Cells 

**Figure 4:**
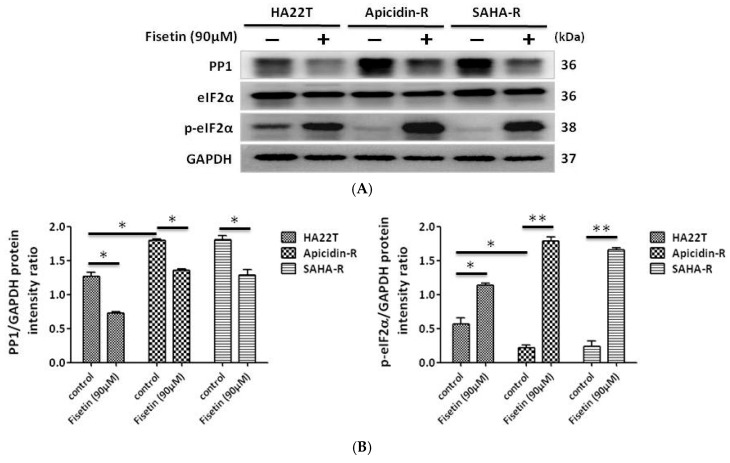
Fisetin-induced eIF-2α phosphorylation in HCC cells via the inhibition of the protein phosphatase 1 (PP1)-mediated regulatory system for eIF2α phosphorylation. HA22T, apicidin-R, and SAHA-R cells were treated with fisetin (90 μM) for 24 h. (**A**) p-eIF2α levels were increased and PP1 expression was decreased in HCC cells upon fisetin treatment. Interestingly, increased PP1 protein expression was noted in apicidin-R and SAHA-R cells compared with HA22T in normal conditions. (**B**) In Fig. 4A, all protein samples were analyzed by western blotting. The protein expression was normalized to GAPDH. * *p* < 0.05, ** *p* < 0.01, *** *p* < 0.001 compared with the control group.

This study demonstrated that fisetin induced the phosphorylation of eIF-2α independent of PERK activation. To explore the molecular mechanism of eIF-2α phosphorylation, we examined the expression levels of PP1. The results indicate that the expression of PP1 protein was increased in HDACis-R cells compared with parental cells in normal conditions. Thus, the high expression of p-eIF-2α in parental cells is due to PP1 inhibition. PP1 expression was significantly reduced and p-eIF-2α expression was increased by fisetin in hepatocellular carcinoma (Figure 4A,B). The results indicate that PP1 inhibition increases the expression of p-eIF-2α.

### 2.5. Protein Phosphatase 1 Is Significantly Engaged in the Effect of Fisetin on HCC Apoptosis

**Figure 5:**
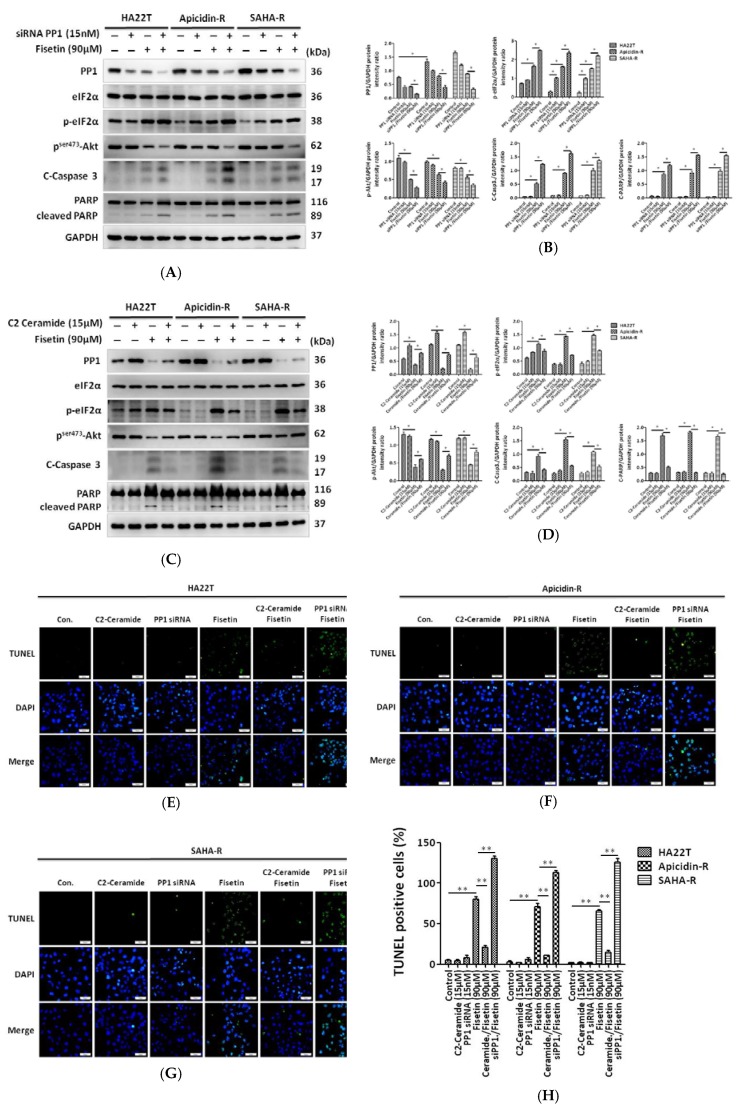
Role of PP1 in the in vitro death of liver cancer cells (**A**,**B**) Downregulation of PP1 expressed by siRNA induced HCC cell apoptosis by fisetin. Western blot quantification using the ImageJ software, normalized to GAPDH. * *p* <0.05, ** *p* <0.01, *** *p* <0.001 compared with the control group. (**C**,**D**) C2 ceramide increased PP1 expression and rescued cell death induced by fisetin. Western blot quantification using ImageJ, normalized to GAPDH. * *p* < 0.05, ** *p* < 0.01, *** *p* < 0.001 compared with the control group. (**E**–**G**) DNA fragmentation was determined by TUNEL assay in HCC cells with PP1 knockdown or C2 ceramide (PP1 activator) treatment with or without fisetin treatment. Upper panel: HCC cells were stained with fluorescein-labeled dUTP according to the protocols reported in the Methods section. Green fluorescence indicates TUNEL-positive cells. Lower panel: HCC cells in the upper panel were stained with 4',6-diamidino-2-phenylindole (DAPI) to identify cell nuclei. Scale bar = 50 μm. (**H**) The apoptosis rate was calculated as the percentage of TUNEL-positive cells among the total number of counted parental and resistant cells (mean ± SD, *n* = 3). The mean values were significantly different compared with the control group. * *p* < 0.05, ** *p* < 0.01, *** *p* < 0.001.

Next, we reconfirm that the PP1-eIF2α pathway is involved in fisetin-induced HCC apoptosis. Our data reveal that not only siRNA but also fisetin downregulate PP1 expression. PP1 siRNA enhanced fisetin-induced apoptosis in HCC cells. Specifically, the expression of the pro-survival protein p-Akt decreased, and the expression of cleaved caspase 3 or cleaved PARP increased after knockdown of PP1 within fisetin (Figure 5A,B). Moreover, treatment with C2 ceramide (15 μM), which is a PP1 activator [40,41], does not affect eIF2α expression in the absence of fisetin in liver cancer cells. However, C2 ceramide rescued fisetin-induced apoptosis by reducing levels of p-eIF-2α, cleaved caspase 3, and cleaved PARP, and increased p-Akt expression in HCC (Figure 5C,D). Using terminal deoxynucleotidyl transferase dUTP nick end labeling (TUNEL) assays, we found that fisetin induced cell apoptosis in HCC cells compared with the control. PP1 siRNA treatment in combination with fisetin significantly increased the number of TUNEL-positive cells compared with the fisetin treatment group. Furthermore, C2 ceramide treatment with fisetin reduced the percentage of apoptotic cells compared with cells treated with fisetin alone (Figure 5E–H). 

### 2.6. Antidrug Effect of p-eIF2α on HDACis-R and Parental Cell Lines

**Figure 6:**
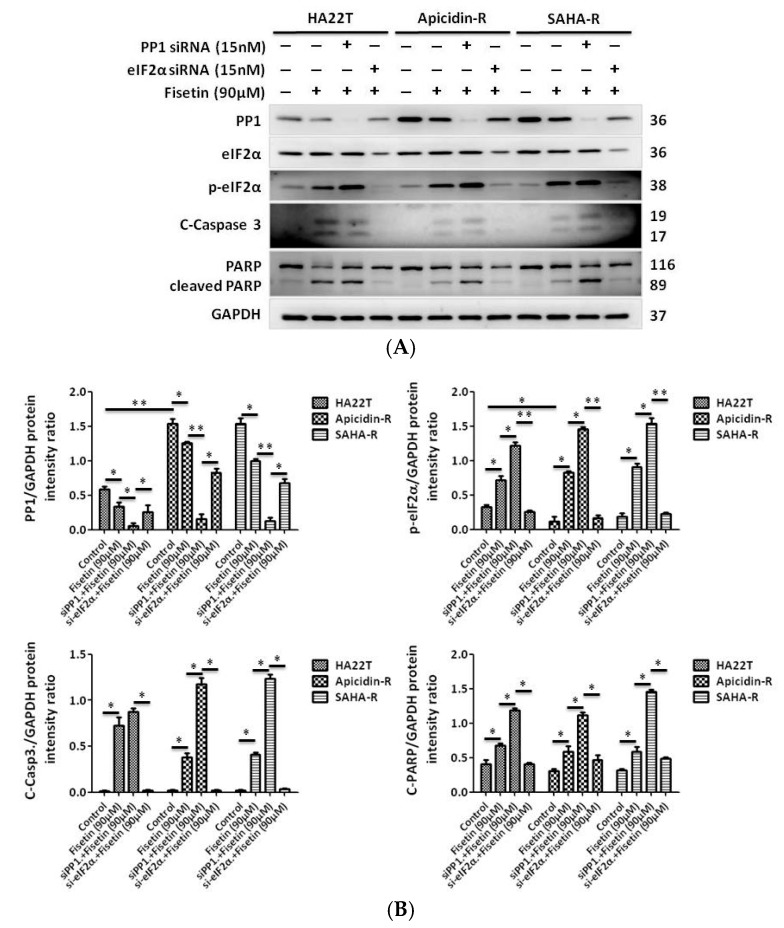

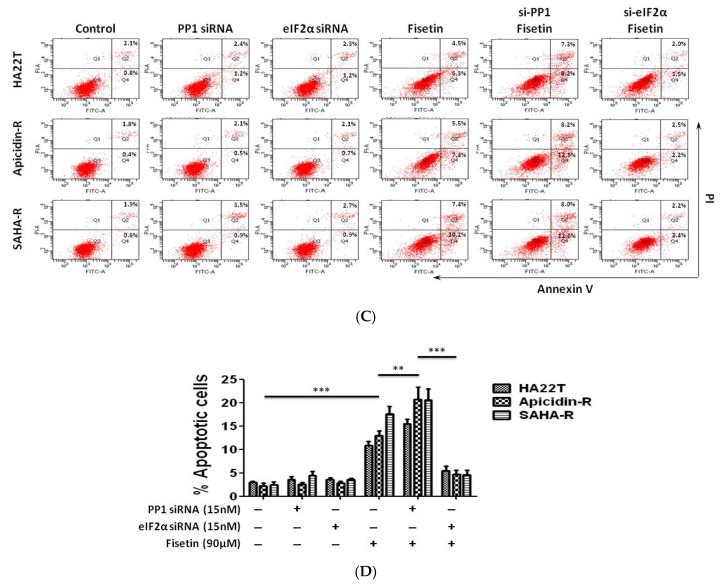
Knockdown of eIF2α by gene silencing inhibits the extraordinary apoptosis effect in HCC. (**A**,**B**) siRNA knockdown of eIF2 expression downregulated p-eIF2α expression, and levels of cleaved caspase 3 or cleaved PARP also decreased, which promoted cell survival. Quantification of western blot using ImageJ, normalized to GAPDH. * *p* < 0.05, ** *p* < 0.01, *** *p* < 0.001 compared with the control group. (**C**) After the regulated expression of PP1 or eIF2α and treatment with or without fisetin to check cell apoptosis in liver cancer cells by flow cytometry. (**D**) Quantification of Figure 6C. Data indicate the percentage of apoptotic cells (annexin-V positive + annexin-V and propidium iodide (PI) double positive cells) (*p < 0.05, ** *p* < 0.01, *** *p* < 0.001) compared with the nontreatment group.

Next, we used eIF2α siRNA to detect the effect of eIF2α phosphorylation in fisetin-treated HCC cells. HCC cells were transfected with eIF2α siRNA for 24 h and treated with 90 μM fisetin for 24 h afterwards. Cell apoptosis was measured by western blot and flow cytometry. Here, eIF2α siRNA-transfected HCC cells treated with fisetin exhibited significantly reduced expression of p-eIF2α, cleaved caspase 3, and cleaved PARP (Figure 6A,B). The same results revealed that fisetin treatment alone with PP1 siRNA treatment induced cell apoptosis in HCC cells compared with the control, as shown by using annexin V and propidium iodide (PI) staining. Importantly, the eIF2α siRNA-transfected cells with fisetin treatment exhibited reduced apoptosis compared with fisetin treatment alone or PP1 siRNA-transfected cells treated with fisetin (Figure 6C,D). These results indicate that eIF2α siRNA transfection reduced fisetin injury and conferred fisetin resistance in human HCC cells. 

### 2.7. Fisetin Enhanced the Therapeutic Potential in Xenograft Tumors Generated from HDAC Inhibitor-Resistant Cells

**Figure 7:**
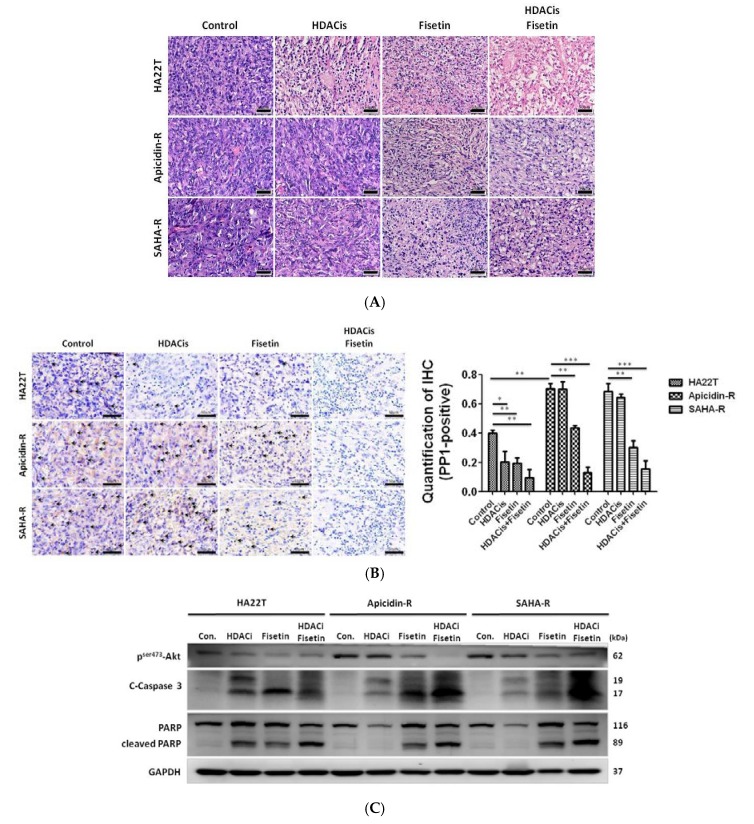

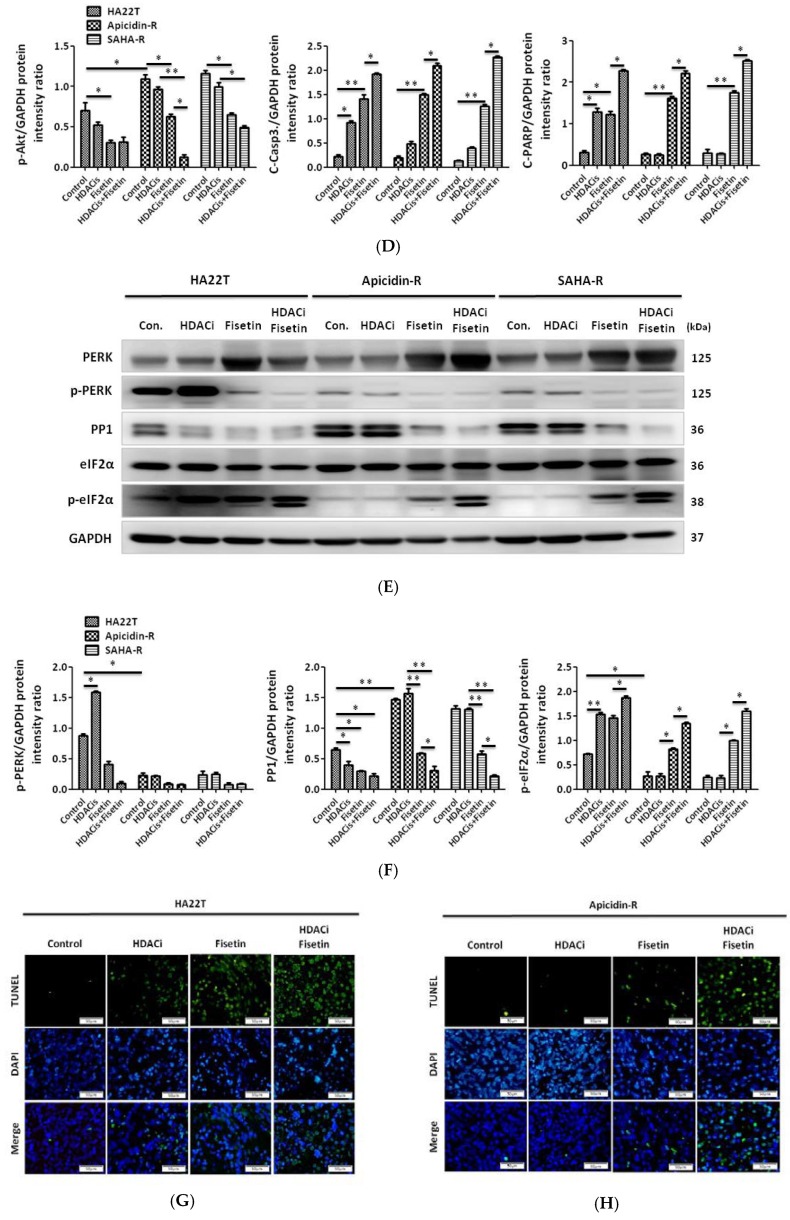

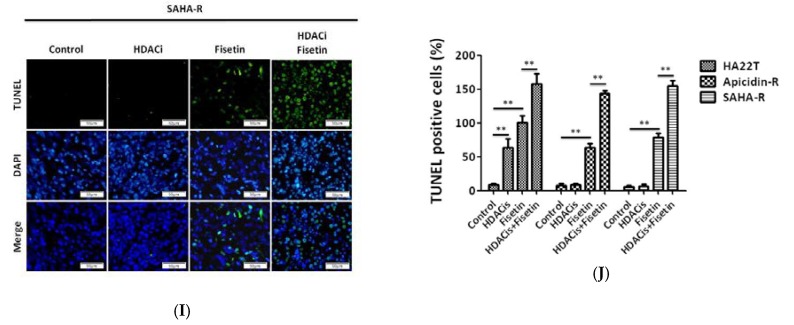
Effect of treatment with fisetin and HDAC inhibitors (apicidin, SAHA), both alone and in combination, on HA22T tumors and HDACis-R tumors in athymic nude mice. Tumors from athymic nude mice implanted with HA22T and HDACis-R cells and treated with the vehicle or fisetin or HDAC inhibitors (apicidin, SAHA) alone or in combination were harvested for immunostaining, Western blot analyses and TUNEL assay. (**A**) Tissue sections stained by hematoxylin and eosin (H&E stain). Scale bar = 50 μm. (**B**) PP1 expression levels (brown color and quantification graph data) in liver tumor samples were examined by IHC analysis. Arrows indicate PP1-immunoreactive cells. Scale bar = 50 μm. (**C**) Western blotting for the cleavage of caspase-3 and PARP and p-Akt expression. The data presented are from a representative experiment repeated thrice with similar results. (**D**) Quantification of Figure 7C, normalized to GAPDH. * *p* <0.05, ** *p* <0.01, *** *p* <0.001 compared with the control group. (**E**) Protein lysates were examined for the expression of proteins in the ER stress sensor PERK and PP1 signaling pathways. The data presented were obtained from a representative experiment repeated thrice with similar results. (**F**) Quantification of Figure 7E, normalized to GAPDH. * *p* < 0.05, ** *p* < 0.01, *** *p* < 0.001 compared with the control group. (**G**–**I**) Sections from tumors harvested from mice treated with vehicle, fisetin, or HDAC inhibitors alone or in combination exhibited DNA damage, as determined by TUNEL assays. Scale bar = 50 μm. (**J**) The apoptosis rate was calculated as the percentage of TUNEL-positive cells among the total number of counted parental and resistant cells (mean ± SD, n = 4). The mean values were significantly different compared with the control group. * *p* < 0.05, ** *p* < 0.01, *** *p* < 0.001.

To precisely monitor the effects of fisetin, HDAC inhibitors, and their combination on the growth of HA22T and HDACis-R cells in nude mice, cells were implanted subcutaneously in 26 mice. After subcutaneous injection for four weeks, we injected 90 μM fisetin, HDAC inhibitors (3 μM SAHA or 10 μM apicidin), and their combination into the tumors for two weeks. Then, mice were sacrificed for analysis. Tissue sections stained by hematoxylin and eosin revealed that HDAC inhibitors resulted in tumor cell disorder in the HA22T group but had no effect in the HDACis-R group. Interestingly, fisetin induced tumor cell disorder in both the HA22T and HDACis-R groups. Additionally, treatment with fisetin alone and the combined treatment with fisetin and HDAC inhibitors induced more cell gap and disorder, especially in the HDACis-R group (Figure 7A). An immunohistochemical (IHC) analysis of tumor sections showed that PP1 expression changed in fisetin- and HDAC inhibitors-treated tumors compared with the respective untreated control groups. Importantly, the reduction of PP1 expression was greater in the combined treatment groups (Figure 7B). Western blotting results indicate that fisetin also induced tumor tissue apoptosis via the cleavage of caspase-3 and PARP and the reduction of the phosphorylation of Akt at Ser473. In particular, the combined treatment (fisetin and HDAC inhibitors) was more effective at inducing apoptosis in HDACis-R tumors compared with the individual agents (Figure 7C,D). Following previous data, we examined the ER stress and PP1 signaling pathways in our animal model. Immunoblotting analysis showed that the combined treatment was more effective in reducing PP1 expression and increasing eIF2α phosphorylation in HA22T tumors compared with either fisetin or HDAC inhibitors alone, especially in the HDACis-R groups (Figure 7E,F). These results highlight fisetin’s ability to inhibit tumor growth and induce apoptosis by modulating the PP1-eIF2 signaling pathway in liver cancer cells in vivo, especially in the HDACis-R groups. To confirm that fisetin enhanced HDAC inhibitors-mediated apoptosis in xenograft tumors, TUNEL assays were performed. The results revealed that HDAC inhibitors induced DNA damage only in the HA22T group, and not in the HDACis-R groups (Figure 7G–J). Fisetin induced apoptosis in the three xenograft tumor groups. Importantly, the combination of fisetin and HDAC inhibitors more significantly induced tumor apoptosis in the HDACis-R groups. 

## 3. Discussion

In this study, we attempted to identify natural extracts that could be used in a supplementary therapy to combat chemoresistance to HDAC inhibitors in HCC cells, in order to produce a potential new treatment method for clinical trials of HDAC inhibitor chemoresistance. We demonstrate that fisetin not only induced HA22T cell apoptosis but also inhibited HDAC inhibitors-resistant cell line growth and proliferation. Importantly, fisetin enhanced chemosensitivity in HDAC inhibitors-resistant cells. The major effect of fisetin on liver cancer cells was the mediation of the PP1/eIF2α pathway, however it also regulated the expression of p-Akt, c-caspase 3, and c-PARP. Importantly, we elucidated a new drug resistance mechanism mediated by the dephosphorylation of eIF2α via PP1 in liver cancer cells resistant to HDAC inhibitors. Furthermore, our results indicate that fisetin could be an adjuvant drug candidate in the context of chemotherapeutic resistance and could facilitate the development of antitumor strategies or combination therapies.

Chemotherapy is a common cancer treatment, and also causes many side-effects and results in chemoresistant tumors in patients [42]. Therefore, alternative cancer treatments are needed. Natural extracts have been reported that have many beneficial effects on human health and regulate apoptosis signaling pathways [43]. Several studies have indicated that the nontoxic dietary flavonoid fisetin possesses anti-tumor properties [44] and is therefore suitable for development as a chemopreventive or chemotherapeutic agent for liver cancer. The objective of this study was to investigate whether the apoptosis and chemosensitivity of HDAC inhibitors-resistant hepatocellular carcinoma cells could be regulated by fisetin, and to determine which molecular mechanisms and signaling pathways were involved. We elucidated the underlying mechanisms by which fisetin enhances the apoptosis and chemosensitivity of HDAC inhibitors-resistant hepatocellular carcinoma cells. We additionally demonstrated that fisetin increases chemosensitivity, leading to cell death in hepatocellular carcinoma cells, possibly by reducing the level of PP1 expression, inducing eIF2α phosphorylation, and promoting apoptosis.

The results of this study show that fisetin alone or in combination with an HDAC inhibitors reagent attenuated liver cancer cell viability in a dose-dependent manner, and thus that fisetin could be a beneficial cancer therapy and a potential treatment for multidrug-resistant cancers.

Many studies have demonstrated that fisetin can inhibit cancer migration and invasion by suppressing the p38 MAPK-dependent NF-κB signaling pathway, causing downregulation of urokinase-type plasminogen activator (uPA) [44] and reducing cancer cell growth and proliferation by inducing apoptosis mediated by ER stress [45]. Thus, ER stress is a frequent cellular response to anticancer treatment. The phosphorylated eIF2α mechanism is a signal pathway which results in apoptosis [46]. In our study, we found that fisetin did not increase levels of phosphorylated eIF2α through the activation of the ER stress sensor protein PERK, but rather through serine/threonine (Ser/Thr) phosphatase protein PP1 inhibition in HA22T and HDACis-R cells. More importantly, we demonstrate that the PP1 activator C2 ceramide enhances drug resistance in HA22T and HDACis-R cells. PP1 is a Mn2+-dependent protein phosphatase with activity towards phosphoserine/threonine residues [47] that is involved in multidrug resistance in human cancers [48]. 

C2 ceramide has been shown to exert potent anti-tumor effects in different cancer types, and may regulate cancer cell apoptosis by autophagy activation [49]. However, research has indicated that treatment with C2 ceramide alone cannot significantly activate autophagy after a combination therapy of chloroquine and C2 ceramide, which enhanced cell apoptosis and autophagy in lung cancer cells [50]. Importantly, previous studies applied C2 ceramide treatment at a dose of 25–50 μM to activate autophagy [51], whereas in this study, we used a dose of 15 μM to regulate PP1 (Figure 5C) activation without autophagy activation (Supplementary Figure S1). Moreover, ceramide regulated multiple downstream targets to regulate autophagy activation, cell apoptosis and phosphatase activation. In our study we used ceramide and PP1 siRNA to confirm whether fisetin regulated cancer cell chemosensitivity via PP1 expression. Importantly, fig. 5C showed after treated with C2 ceramide 15 μM induced PP1 expression without cleaved caspase 3 and PARP expression. Accordingly, we treated with low-dose C2 ceramide to activate PP1 without cell apoptosis and autophagy activation and siRNA to confirm PP1 play an important role in the chemosensitivity of liver cancer cells and HDAC inhibitors resistance cells. 

**Figure 8:**
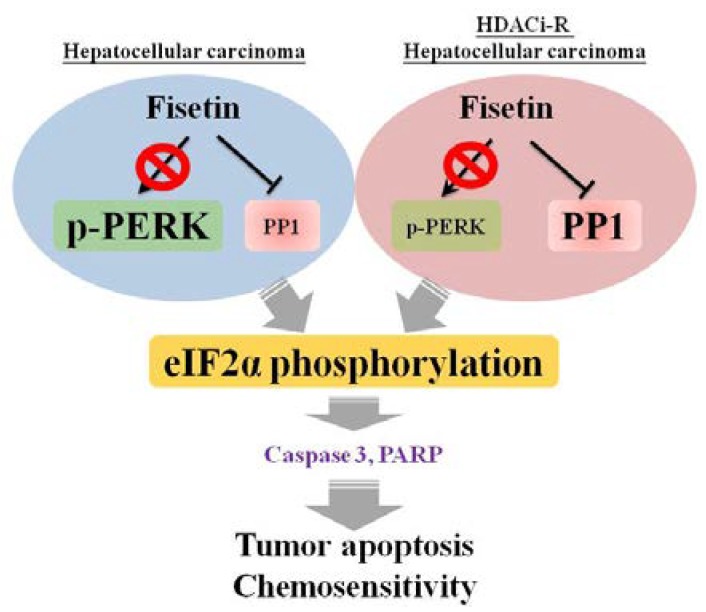
Schematic representation of fisetin-induced chemosensitivity via p-eIF2α and the inhibition of PP1 in HCC cells.

Importantly, we demonstrated the critical role of PP1/p-eIF2α in HCC tumorigenesis and chemosensitivity. In this study, we found that PP1 was highly expressed in HDACis-R cells, and that treatment with fisetin reduced PP1 expression, upregulated p-eIF2a expression, and induced cell apoptosis. These results showed that fisetin induces cell apoptosis, possibly through eIF2α phosphorylation via PP1 (Figure 8).

## 4. Materials and Methods 

### 4.1. Cell Culture and Establishment of Resistant Cells

Human hepatocellular carcinoma cell line (HA22T, Asia Bioscience Co., Ltd., Taipei, Taiwan, GRL-CLC011) and normal rat liver epithelial cell line (Clone 9, ATCC CRL1439) cells were cultivated in Dulbecco’s minimum essential medium (DMEM; Sigma, St. Louis, MO, USA, Cat. No. D5523) with 1% penicillin (Corning, Corning, New York, NY, USA, Cat. No. 30-002-CI) and 10% fetal bovine serum (HyClone, Long, UT, USA, Cat. No. SH3007103) at 37 °C in a humidified atmosphere of 5% CO_2_ and 95% air. In our previous studies, apicidin-resistant cells (apicidin-R cells) were established from HA22T cells that were exposed to increasing concentrations of apicidin [10,20]. It was also demonstrated that apicidin-R cells are resistant to SAHA [22], and apicidin-R cells have been approved by the U.S. Food and Drug Administration (FDA) for the treatment of patients with CTCL and show promise in treating patients with HCC. In this study, SAHA-R cells were established from apicidin-R cells which were exposed to increasing concentrations of SAHA.

### 4.2. Whole-Cell Extraction

Cellular protein was extracted using RIPA lysis and extraction buffer (Thermo Fisher Scientific, Waltham, MA, USA) containing a protease k inhibitor and phosphatase inhibitor. Cell pellets were lysed for 30 min, and samples were centrifuged at 12,000 rpm for 20 min at 4 °C in a microcentrifuge. The supernatant liquid was collected in new Eppendorf tubes and stored at –20 °C.

### 4.3. Cell Viability Assay

Cell viability was measured using the MTT assay, which is based on the conversion of yellow tetrazolium MTT (3-(4,5-dimethylthiazolyl-2)-2,5-diphenyltetrazolium bromide) into formazan crystals using mitochondrial dehydrogenase enzymes. Briefly, cells were plated in triplicate in 24-well plates and treated with increasing concentrations of fisetin, apicidin, and SAHA alone or in combination. After 24 h or 48 h of incubation, 0.5 mg/ml of MTT (Sigma-Aldrich Inc., St Louis, MO, USA) was added to each well, and the samples were incubated at 37 °C in a humidified atmosphere of 5% CO_2_ and 95% air for 2–4 hours until purple precipitate was visible. The purple MTT formazan precipitate was dissolved in 200 μl of DMSO and the absorbance of the formazan was measured at 570 nm using an automated microplate reader (Thermo Fisher Scientific). The CI was calculated using the CompuSyn software (ComboSyn Inc., Paramus, NJ, USA). The CI is a quantitative measure of the degree of drug interaction—CI < 1 indicates synergism, CI = 1 indicates an additive effect, and CI > 1 indicates antagonism [37].

### 4.4. Antibodies and Drug formulations

The following antibodies were used in this study: anti-PERK (sc-377400, Santa Cruz, CA, USA), anti-p-PERK Thr981 (sc-32577, Santa Cruz, CA, USA), anti-eIf2α (sc-133132, Santa Cruz, CA, USA), anti-PP1 (sc-7482, Santa Cruz, CA, USA), anti-p-Akt1/2/3 (sc-7985, Santa Cruz, CA, USA), anti-GAPDH (sc-32233, Santa Cruz, CA, USA), anti-cleaved-caspase 3 (#9664, Cell Signaling Technology, Danvers, MA, USA), anti-p-eIF2α Ser51 (#9721, Cell Signaling Technology), and anti-PARP (#9542, Cell Signaling Technology). All the secondary antibodies (HRP-conjugated anti-rabbit, anti-mouse, and anti-goat) and all the drug formulations were purchased from Sigma-Aldrich Inc.

### 4.5. Western Blot Analysis

Total protein from mammalian tissue or cell samples was extracted with cell lysis buffer (mixed 50 mM Tris–HCl, 2.5 mM DTT, 0.1% Triton X-100, 2.5 mM EDTA, 5 mM imidazole, 250 mM sucrose, and adjusted to a pH of 7.4) and spun down at 12,000 rpm for 20 min. The protein concentration was measured using the Bradford protein assay (Bio-Rad Laboratories Inc., Hercules, CA, USA). Subsequently, an aliquot of each sample extraction equivalent to 20 μg protein was boiled after addition of the appropriate amount of 5-fold sample buffer (mixed 162 mM, 5% SDS, DTT5 mM EDTA, 0.5 mL bromophenol blue, 50% glycerol, 188 mM Tris, and adjusted to a pH of 8.8). The proteins were separated using 8–12% SDS-PAGE and transferred to PVDF membranes (Millipore, Belford, MA, USA) using the Bio-Rad electrotransfer system (Bio-Rad Laboratories Inc.). The membranes were blocked with blocking buffer (mixed 5% non-fat milk, 150 mM NaCl, 0.1% Tween-20 and 20 mM Tris-HCl and adjusted to a pH of 7.6) for 1–2 h at room temperature and probed with specific primary antibodies at 4 °C overnight. Then, the protein bands were measured with HRP-conjugated secondary antibodies for 1 h at room temperature using custom-made ECL (Millipore, Billerica, MA, USA) detection reagents. An ImageQuant LAS 4000 digital imaging system (Digital Image Systems, Commerce, CA, USA) was used to detect the target protein on immunoblot PVDF membranes.

### 4.6. TUNEL assay and DAPI Staining

Cell apoptosis was detected by terminal transferase-mediated dUTP nick-end labeling of nuclei using the TUNEL Assay Kit (Roche Ltd., Basel, Switzerland) following the manufacturer's protocol. Liver cancer cells were inoculated into chamber slides, fixed by 4% paraformaldehyde for 20 min and permeabilized with 0.1% Triton X-100 in 0.1% sodium citrate for 10 min. The cellular nuclei were stained with the TUNEL Assay Kit overnight at 4 °C, and then DAPI (blue) (Sigma-Aldrich Inc.) was added for 10 min. The TUNEL-positive cells (green) and DAPI (blue) staining patterns were detected by fluorescence microscope (Olympus, Tokyo, Japan). Moreover, liver cancer tissue (3 μm thickness) was placed on slides and stained with the TUNEL Assay Kit. To estimate the number of apoptotic cells among the liver tumor cells, digital images were acquired at ×200 magnification, and three different fields from each sample were quantified.

### 4.7. FITC-Annexin V Staining for Apoptosis

Cell apoptosis was assessed using the FITC-Annexin V Apoptosis Detection kit I (BD Biosciences, San Jose, CA, USA). In brief, cell apoptosis was induced by the desired method and then cells were collected and washed twice with PBS. Then, 1 × 10^6^ cells were resuspended in 1 ml of 1× binding buffer. After that, a suspension of 1 × 10^5^ cells was transferred to a 5 ml test tube, 5 μL of annexin V and 5 μL of PI were respectively added, and the suspension was gently vortexed and incubated at room temperature for 15 min in the dark. Then, 1× binding buffer was added to make the total volume 500 μL for each tube. Finally, cell apoptosis was analyzed by flow cytometry within 1 h.

### 4.8. Animal Model

Six-week-old male NU/NU nude mice were obtained from BioLASCO Taiwan Co., Ltd. (Taipei, Taiwan) and fostered following the China Medical University Institutional Animal Care and Use Committee of guidelines. The animal use protocol listed below has been reviewed and approved by the Institutional Animal Care and Use Committee in China Medical University (protocol code: 2016-172-1). The mice were divided into 12 groups (n = 4 each): three control groups (subcutaneous injection of HA22T cancer cells, apicidin-R cells, SAHA-R cells), three controls with tumors injected with HDAC inhibitors (SAHA and apicidin), three controls with tumors injected with fisetin, and three controls with tumors injected with HDAC inhibitors and cotreated with fisetin. The number of cancer cells used for subcutaneous injection in the hind legs of the nude mice was 1 × 10^7^ cells suspended in Matrigel matrix with 50% serum-free DMEM. Prior to further treatment, mice were maintained until tumors reached 500 mm^3^. After the eight-week experimental period, the mice were sacrificed, and the tumors were removed and weighed. A portion of the tissue was fixed in 10% formaldehyde for histopathological analysis and the other residual portion was stored at –80 °C for further biochemical measurements. 

### 4.9. Histopathological Examination

The tumor tissue was fixed using 10% buffered formalin to inhibit autolysis or decay and further analysis of histological alterations was performed. The processing procedure involved dehydration, clearing of fixative, and embedding in paraffin wax. To study the tumor histology of nude mice, liver cancer tissue slides (3 μm thickness) were observed under light microscopy at ×200 magnification (OLYMPUS BX53, Olympus, Tokyo, Japan) (scale bar 50 µm) after staining with hematoxylin and eosin.

### 4.10. Immunohistochemistry

The 3 μm thick liver tumor slides were individually treated using xylene for dewaxing and were further treated using a staining protocol with horseradish peroxidase-conjugated avidin biotin complex (ABC) from the Vectastain Elite ABC Kit (Vector Laboratories, Burlingame, CA, USA) and NovaRed chromogen (Vector Laboratories) and counterstained with hematoxylin. The PP1-positive staining was detected in the cytoplasm using a microscope (OLYMPUS BX53) and quantification using the ImageJ software (NIH, Bethesda, MD, USA).

### 4.11. Statistical Analysis

Statistical analysis was performed by one-way analysis of variance (one-way ANOVA) using the SigmaPlot 10.0 software (Systat Software Inc., San Jose, CA, USA) with GraphPad Prism 8. In all tests, the P-values were less than 0.05, and were regarded as statistically significant, while the values ** *p* < 0.01 and *** *p* < 0.001 were considered to indicate increased statistical significance.

## 5. Conclusions

This investigation of fisetin-induced liver cancer cell death and enhanced liver cancer cell chemosensitivity both in vitro and in vivo, especially in HDACis-resistant cells, suggests that fisetin may serve as an adjuvant treatment for chemotherapy in cancer treatment.

## Supplementary Materials

The following are available online at https://www.mdpi.com/2072-6694/11/7/918/s1, Figure S1. To check whether treated with C2 ceramide for 24 h affect autophagy activation in liver cancer cells.
