# Correction: Liu, Y.-S.; et al. Inhibition of Protein Phosphatase 1 Stimulates Noncanonical ER Stress eIF2α Activation to Enhance Fisetin-Induced Chemosensitivity in HDAC Inhibitor-Resistant Hepatocellular Carcinoma Cells. *Cancers* 2019, *11*, 918

**DOI:** 10.3390/cancers12113241

**Published:** 2020-11-03

**Authors:** Yi-Sheng Liu, Yu-Chun Chang, Wei-Wen Kuo, Ming-Cheng Chen, Hsi-Hsien Hsu, Chuan-Chou Tu, Yu-Lan Yeh, Vijaya Padma Viswanadha, Po-Hsiang Liao, Chih-Yang Huang

**Affiliations:** 1 Program for Aging, China Medical University, Taichung 404, Taiwan; menace0712@yahoo.com.tw; 2 Division of Hematology and Oncology, Department of Medicine, Kaohsiung Armed Forces General Hospital, Kaohsiung 802, Taiwan; 3 Graduate Department of Biological Science and Technology, National Pingtung University of Science and Technology, Pingtung 912, Taiwan; dye60136@gmail.com; 4 School of Chinese Medicine, China Medical University, Taichung 404, Taiwan; 5 Department of Biological Science and Technology, China Medical University, Taichung 404, Taiwan; wwkuo@mail.cmu.edu.tw; 6 Division of Colorectal Surgery, Department of Surgery, Taichung Veterans General Hospital, Taichung 407, Taiwan; claudiochen7@gmail.com; 7 Faculty of Medicine, National Yang-Ming University, Taipei 112, Taiwan; 8 Division of Colorectal Surgery, MacKay Memorial Hospital, Taipei 104, Taiwan; hsu5936@ms3.hinet.net; 9 MacKay Medicine, Nursing and Management College, Taipei 112, Taiwan; 10 Division of Chest Medicine, Department of Internal Medicine, Armed Force Taichung General Hospital, Taichung 411, Taiwan; tu4697@gmail.com; 11 Department of Pathology, Changhua Christian Hospital, Changhua 500, Taiwan; 1867@cch.org.tw; 12 Department of Medical Technology, Jen-Teh Junior College of Medicine, Nursing and Management, Miaoli 356, Taiwan; 13 Department of Biotechnology, Bharathiar University, Coimbatore 641046, India; vvijayapadma@rediffmail.com; 14 Graduate Institute of Basic Medical Science, China Medical University, Taichung 404, Taiwan; 15 Cardiovascular Research Center, Hualien Tzu Chi Hospital, Hualien 970, Taiwan; 16 Center of General Education, Buddhist Tzu Chi Medical Foundation, Tzu Chi University of Science and Technology, Hualien 970, Taiwan; 17 Department of Medical Research, China Medical University Hospital, China Medical University, Taichung 404, Taiwan; 18 Department of Biotechnology, Asia University, Taichung 413, Taiwan

It has been reported that two sets of blot figures in Figure 3A,C looked identical in the original published paper [1]. After a careful check, the authors found that the p-eIF2α band in Figure 3C was mistakenly uploaded, while the other bands are correct. The authors state this was due to carelessness.

With the approval of the Editor-in-Chief and the Editorial Office, the authors wish to replace Figure 3C in [1] with an updated version. 

The original version of Figure 3C is as follows:
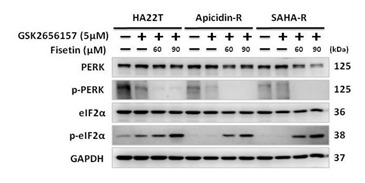
 and should be replaced with the following version:
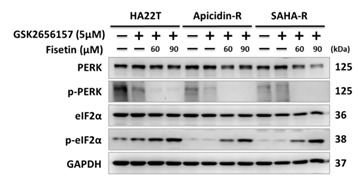


The authors apologize for any inconvenience caused and state that the scientific conclusions are unaffected. The original article has been updated. 
