# Supplementary material for: Inhibition of Protein Phosphatase 1 Stimulates Noncanonical ER Stress eIF2α Activation to Enhance Fisetin-induced Chemosensitivity in HDAC Inhibitor-resistant Hepatocellular Carcinoma Cells
Source: Cancers (Basel). 2019 Jun 29;11(7):918. doi: 10.3390/cancers11070918 (PMC6678694; doi:10.3390/cancers11070918)
Supplement: Supplementary file 1 [file cancers-11-00918-s001.pdf]

Supplementary Materials

Inhibition of protein phosphatase 1 stimulates noncanonical ER stress eIF2α activation to enhance fisetin-induced chemosensitivity in HDAC inhibitor-resistant hepatocellular carcinoma cells

Yi-Sheng Liu, Yu-Chun, Chang, Wei-Wen Kuo, Ming-Cheng Chen, Hsi-Hsien Hsu, Chuan-Chou Tu, Yu-Lan Yeh, Vijaya Padma Viswanadha, Po-Hsiang Liao and Chih-Yang Huang

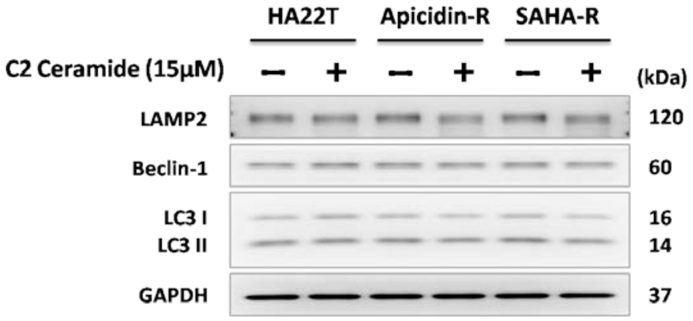

Figure S1. To check whether treated with C2 ceramide for 24 h affect autophagy activation in liver cancer cells. After treated with C2 ceramide 15 μM cannot activate autophagy in liver cancer cells determined by western blotting.

© 2019 by the authors. Licensee MDPI, Basel, Switzerland. This article is an open access article distributed under the terms and conditions of the Creative Commons Attribution (CC BY) license (http://creativecommons.org/licenses/by/4.0/).
